# Fabrication of Three-Dimensional Microvalves of Internal Nested Structures Inside Fused Silica

**DOI:** 10.3390/mi12010043

**Published:** 2021-01-01

**Authors:** Chao Shan, Qing Yang, Hao Bian, Xun Hou, Feng Chen

**Affiliations:** 1 State Key Laboratory for Manufacturing System Engineering and Shaanxi Key Laboratory of Photonics Technology for Information, School of Electronic Science and Engineering, Xi’an Jiaotong University, Xi’an 710049, China; shanchao@xjtu.edu.cn (C.S.); haobian@mail.xjtu.edu.cn (H.B.); houxun@mail.xjtu.edu.cn (X.H.); 2 School of Mechanical Engineering, Xi’an Jiaotong University, Xi’an 710049, China; yangqing@xjtu.edu.cn

**Keywords:** microvalve, femtosecond laser, microfluidic, microstructure fabrication

## Abstract

Nested structures inside the hard material play a pivotal role in the microfluidics systems, such as the microvalve and the micropump. In this article, we demonstrate a novel and facile method of fabricating nested structures inside the fused silica with a two-step process femtosecond laser wet etching (FLWE) process. Inside fused silica, a spherical structure was made with a diameter of nearly 80 µm in a square chamber. In addition, we designed a simple microvalve with this sphere controlling the current’s flow. The novel microvalve structure can be easily integrated into the functional microfluidics systems and will be widely applied in the Lab-on-chip (LOC) system.

## 1. Introduction

The three-dimensional (3D) spherical structure nested in the internal microcavity of fused silica has wide applications. Many novel micro-nano devices have been fabricated with this structure [1,2,3]. For example, the nested microsphere can be fabricated into a movable plug to realize a microsyringe, which can precisely control the amount of liquid injected. The microsyringe can be widely used in biomedicine. The 3D structure can also be fabricated into microsensors, such as microgyroscopes and microaccelerometers, by some fabrication methods [4]. It can be well integrated into microfluidic systems to improve the miniaturization and integration performance of microfluidic chips and can be applied in the field of microsatellites or biochips.

Among these micro-nano devices, microvalve structures can control liquid flow driven by a current signal, so it becomes an important control unit in the microfluidic devices [5,6,7]. The existing traditional microvalves are generally assembled by a cavity and a valve plate [8,9,10]. The fabrication of the cavity and valve plate requires high precision. The precision of the assembly is also very strict, which greatly increases the preparation cost and technical difficulty of a microvalve. Besides, due to the limitations of the valve plate itself, the size of the fabricated microvalve is relatively larger, the level of integration is lower, and the service life is often shorter. In addition, metal or organic materials are often used in the components of a traditional microvalve, which increases the manufacturing cost of microvalves. The materials even contain some toxic elements, so that its application in the field of the biochip is limited [11].

How to use the micro-nano machining method to fabricate nested structures inside fused silica, a hard and brittle material with good biocompatibility, has become an urgent problem to be solved. In traditional carving technology, the nested structure is often processed. In the processing of stone carving or other arts and crafts, a chisel is used to cut off the excess part of the stone layer by layer to gradually get the desired shape. In addition, in our previous study [12,13,14], we proposed femtosecond laser wet etching technology, which uses a femtosecond laser to modify the material and hydrofluoric acid to remove the modified area for corrosion. This technique can be used to fabricate hollow 3D microchannel structures in fused silica. Inspiried by traditional carving methods, combined with femtosecond laser wet etching technology, this paper proposes a “micro carving craft” in micro-nano manufacturing. By using a femtosecond laser to ablate material layer by layer and then using hydrofluoric acid to carry out directional corrosion, the area that is not ablated by the laser can be preserved, and the microcavity and 3D nested microstructure can be obtained. Compared with the traditional microvalve, the microvalve size fabricated by this method can generally be in the tens of microns, which is far smaller than with traditional microvalves [15]. The smaller microvalve structure can be well integrated with other microfluidic devices, which conforms to the development direction of microfluidic chip miniaturization and integration. Moreover, the cavity and the nested microsphere of the new microvalve fabricated by femtosecond laser wet etching technique can avoid the assembly difficulty and error caused by the separate preparation of the substrates, the diaphragm, and the upper substrates of the traditional microvalve. The fabrication process is relatively simple.

By using the improved femtosecond laser wet etching (FLWE) method, this paper provides a new design and fabrication idea of the microvalve and produces a 3D microsphere structure in which the fused silica, which can move freely in the microcavity structure. The microvalve structure on the micron scale is fabricated in transparent quartz glass, which can be well connected with other microdrive unit microfluidic devices with a high degree of integration. The fabrication of microvalve structure expands the application field of femtosecond laser wet etching technology and also provides a new idea and method for the preparation of complex 3D microfluidic chip devices.

## 2. Materials and Methods

The microvalve manufacturing process includes two steps: the laser writing process and the chemical wet etch. The femtosecond laser micromachining system used to fabricate the microvalve involves a femtosecond laser source (wavelength: 800 nm, pulse duration: 50 fs, repetition rate: 1 kHz), a microscope objective (NA = 0.9, 100×, Nikon, Tokyo, Japan), a programmable three-axis stage (H101A ProScan II Upright Stage, Prior Scientific, Cambridge, UK), a charge-coupled device camera, and a laser beam control system. The microvalve was fabricated nested in the fused-silica substrate (1.0 cm × 1.0 cm × 1.0 mm). The scan line was written by the laser by moving the 3D stage along the pattern path at a speed of 100 μm/s. The laser power was adjusted from 4 mW to 6 mW by a computer-controlled attenuator with temporal modulation of the power compensation. Because when the internal depth difference is 100 μm, the average power is reduced from 100% to 70%. The side length of the microcavity and the diameter of the microsphere structure can be controlled by a computer.

**Figure 1:**
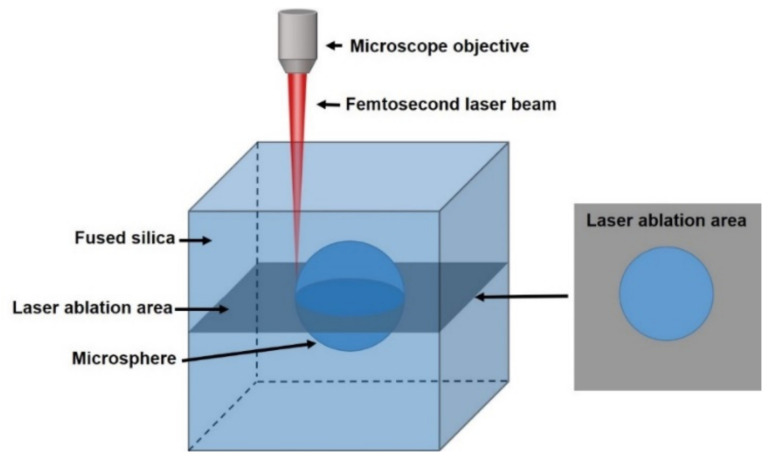
A schematic diagram of laser processing (the gray part is the femtosecond laser ablation area).

Laser scanning was carried out in a layered manner, as shown in Figure 1, with a spacing of 10 μm between layers. In Figure 1, for each layer of scanning, the processing area is gray, and the microsphere area is retained without ablation. The laser power decreases with the decrease of machining depth to ensure the uniformity of machining. The scanning sequence is bottom-up to avoid the blocking and influence of the ablative area on the light. Moreover, we also refer to “the side channel method” of microchannel processing technology and write the channel used to import the corrosion solution directly at the four corners of the ablation area. Second, we used hydrofluoric acid (HF) for wet etching. The microvalve sample was immersed in an ultrasound-assisted solution of 5% for 3 h. A microsphere structure with a dimension of 60 μm inside microcavity was obtained.

## 3. Results

In order to obtain a complete microcavity and microsphere structure with good morphology, the influence of the wet etching process on the processed structure was studied. Firstly, the influence of hydrofluoric acid (HF) concentration on etching reaction is studied. When the concentration of the HF solution increased, the reaction rate with silica also increased. The etching rate can be increased by using a higher concentration of hydrofluoric acid. However, the surface of the microstructures etched by hydrofluoric acid solution with a concentration of more than 15% at room temperature is rough. Moreover, the high concentration is likely to cause an uncontrollable etch rate, which will lead to serious inhomogeneity in microcavity etching and the appearance of cavity structure due to etching of microsphere structure. Finally, after a comprehensive consideration of etch rate, processing structure quality, and other factors, we chose hydrofluoric acid with 5% concentration for the wet etching process. Not only does this ensure etching efficiency, but also ensures the uniformity and surface quality of microcavity and microsphere structure.

**Figure 2:**
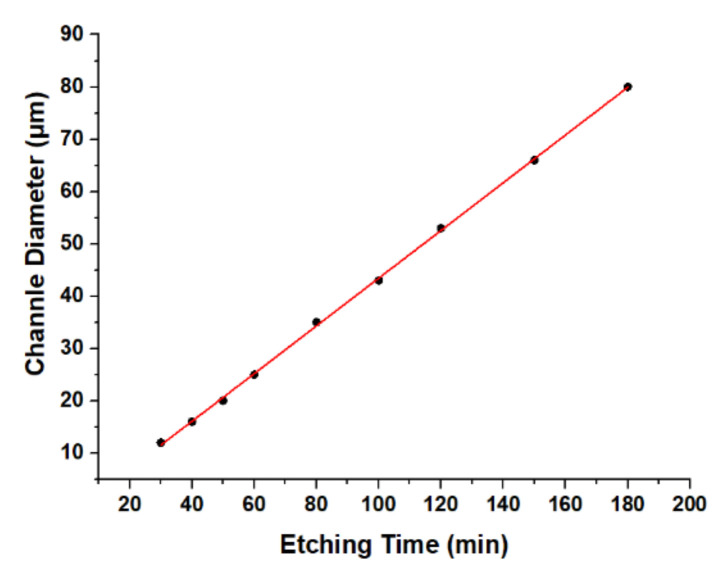
Relation between microchannel diameter and etching time.

Next, we investigated the relationship between the etching time and the etching degree of the material. We chose to use a 6 mW femtosecond laser to scan the linear microchannel inside fused silica at a scanning rate of 100 µm·s^−1^, and etched it with 5% HF solution in the ultrasonic environment. The results are shown in Figure 2.

The results show that the channel diameter increased linearly with the etching time during the process. The sample was periodically removed from the corrosion solution. Therefore, the size of the microcavity and microsphere structure could be controlled by controlling the etching time.

**Figure 3:**
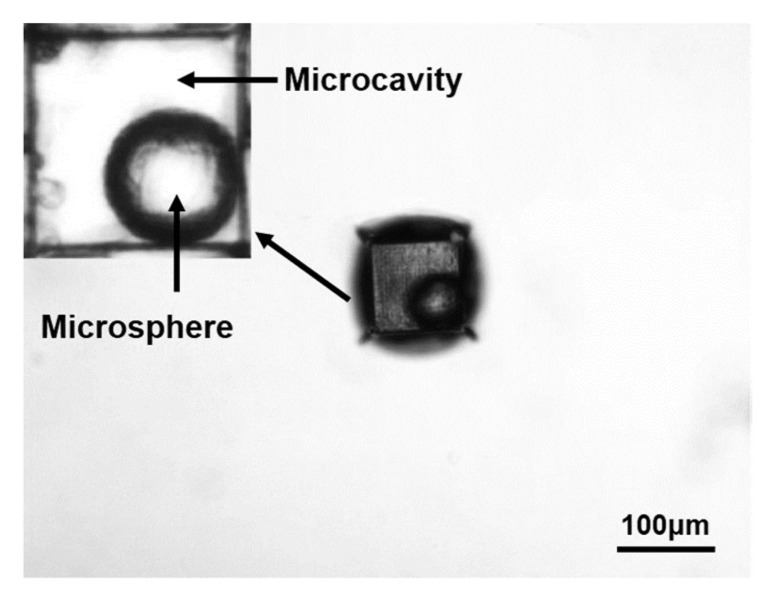
The microcavity and microsphere nested structure inside fused silica.

Using the above experimental method, we first fabricate the nested structure of the microcavity with the microsphere inside the fused silica, as shown in Figure 3 taken by a light microscope, where the diameter of the microsphere was 60 μm and the side length of the microcavity was 100 μm × 100 μm × 100 μm.

## 4. Discussion

Although the microcavity and microsphere nested structure was fabricated in fused silica, the processing results show there were several cracks in the edges and corners of the microcavity. These defects may affect the function of the microvalve structure. Therefore, this problem needs to be solved. First, we analyzed the cause of the crack. When the laser acted on the surface of fused silica, the material was ejected in the plasma state due to the Coulomb microburst. The shock wave was caused by the compression of air due to the rapid expansion of the plasma. As the shock wave pushed outward, its counteracting force exerted great mechanical pressure on the material, resulting in a change of material density. When a femtosecond laser is focused on the fused silica, multiple shockwave effects are formed inside the fused silica [16,17]. The first layer of impact waves is caused by a thermoelastic wave. When a laser is applied to a material, the production and acceleration of large numbers of hot electrons cause them to transfer energy (heat) to surrounding atoms (lattices). As this process is completed within the extreme time order of picoseconds (ps), a sharp rise in local temperature on the surface of the material causes thermal stress to the interior of the material. The shock wave is formed when thermal stress compresses the material. The thermoelastic stress shock wave produced by femtosecond laser acting on quartz glass can reach a pressure of 10^11^ Pa, higher than the modulus of fused silica (10^10^ Pa) [18]. The pressure is strong enough to cause a phase change in the glass to produce a dense region, forming a second and third layer of impulse waves inside the fused silica. The stress wave effect made the actual damaged area in the material larger than the laser spot action area, while the invisible damage range of the high-strength stress wave to the material structure was even larger.

**Figure 4:**
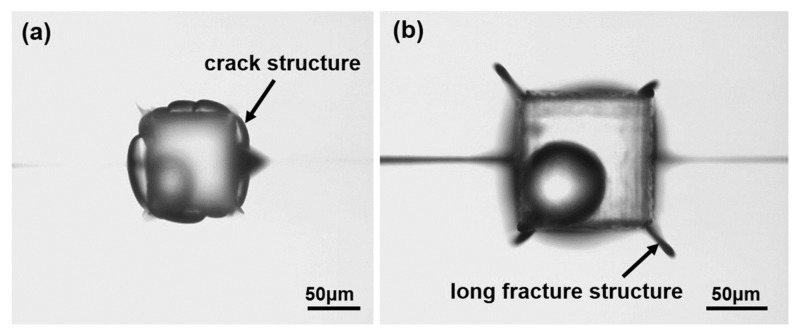
Microcavity nested structure with cracks. (**a**) crack structure with random shape; (**b**) long fracture structures at the four sharp angles.

So, when the femtosecond laser scans in a tiny area, it deposits a huge amount of energy inside the fused silica and creates huge internal pressure. The thermal shock wave and pressure shock wave effect and modify the unprocessed materials around the micro-cavity, and change the structure of the surrounding materials and generate internal stress. When the femtosecond laser modification process is completed, the internal stress and the damaged structure around the fused silica inner microcavity do not disappear but continue to remain inside. After wet etching with the hydrofluoric acid solution, the unablated area around the microcavity was easily corroded by hydrofluoric acid due to internal stress and structural modification. Finally, a crack structure with random shape appeared, as shown in Figure 4a.

On the other hand, we also found that the stress wave was more intense at the corner of the microcavity, and the corner is easy to cause the stress wave caused by femtosecond laser ablation to propagate along a certain direction, similar to the tip effect, resulting in the stress concentration phenomenon. The intensity of the stress wave “concentrated” in this way can increase a lot, which caused deeper damage to the unablated area outside the microcavity. The structure of the microspheres nested in the microcavity is shown in Figure 4b. It can be clearly seen from the figure that long fracture structures appear at the four sharp angles of the microcavity.

In order to avoid the occurrence of cracks in the processing, we have adopted a variety of improvement schemes. Reducing laser power and increasing scanning speed can reduce the number of laser pulses received per unit area in the ablation area, thus reducing the energy density of laser deposition and the stress wave excited. On the other hand, in the design process of the microcavity, it is necessary to avoid the appearance of “sharp angle” and other structures. The microcavity joint adopts the rounded angle structure, which can effectively avoid the stress concentration.

**Figure 5:**
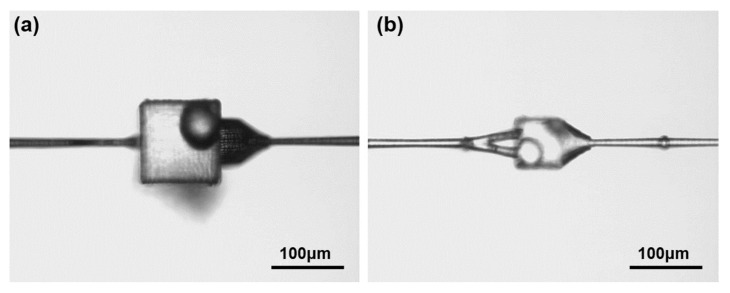
Microvalve device added with attachment structure. (**a**) a micro-cavity device composed of a cylinder and a cone; (**b**) a micro-cavity device composed of a conical structure and excessive channel.

By adopting the improved processing method, we prepared two microdevices of microcavity with microsphere structure by using the processing parameters of 2 mW laser power and 600 μm/s laser scanning speed and combined with the modified processing control program, as shown in Figure 5. These images are a focal plane image of different samples under a light microscope. The laser power density is reduced from 4 mJ/cm^2^ to 0.33 mJ/cm^2^. As shown in Figure 5a, a micro-cavity device composed of a cylinder and a cone was added at the right end. Figure 5b adds a conical structure on the right side of the microcavity and on the left side adopts two simple microdevices connected by an excessive channel at a certain angle. It can be seen from the processing results that there is basically no crack around the microcavity in these two kinds of microdevices.

**Figure 6:**
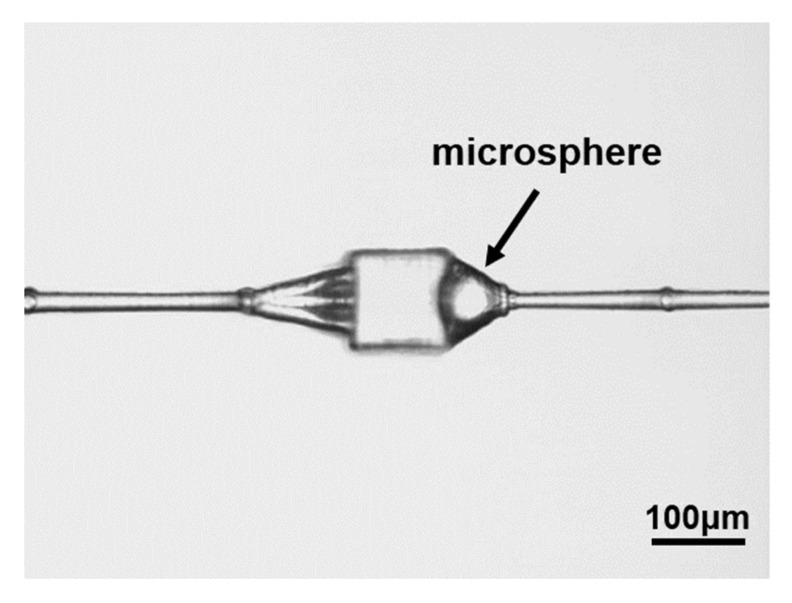
Improved simple microvalve device.

Finally, on the basis of this research, we designed and prepared a 3D microvalve structure, which added a pyramidal structure on the right side of the microcavity to increase the contact between the cavity wall and the microsphere, and connected to the left side with three transition microchannels with a certain angle, as shown in Figure 6. There was no crack structure around the microvalve cavity, and the morphology was good. The structure realized the single guide of the liquid in the microchannel. When the liquid flows from left to right, it will drive the microsphere structure into the cone structure, and blocks the right port, so that the liquid cannot flow to the right anymore. When the liquid flows from right to left, the microsphere structure will move to the left of the microcavity, but its size will be insufficient to simultaneously block three transition microchannels, which will not impact the ability of the liquid to continue to flow to the left. This forms a single guide microvalve structure device.

## 5. Conclusions

In summary, by means of FLWE technology, a microvalve of microcavity and microsphere nested structure can be achieved inside fused silica. Compared to existing microvalve devices, the size of the microvalve device we fabricated has been reduced greatly. This work provides a potential route of realizing high-quality microvalve devices inside fused silica glass and would be beneficial for developing miniaturization and highly integrated microfluidics devices, demonstrating the flexibility and universality of FLWE technology. Meanwhile, a broad spectrum of microfluidics systems fabricated based on these compact and 3D microvalve devices shows promise.
